# Genome-wide analysis of blueberry B-box family genes and identification of members activated by abiotic stress

**DOI:** 10.1186/s12864-023-09704-8

**Published:** 2023-10-03

**Authors:** Xiaoming Liu, Wenying Sun, Bin Ma, Yan Song, Qingxun Guo, Lianxia Zhou, Kuishen Wu, Xinsheng Zhang, Chunyu Zhang

**Affiliations:** 1 College of Plant Science, Jilin University, Changchun, 130062 China; 2 College of Animal Science, Jilin University, Changchun, 130062 China

**Keywords:** B-box, Blueberry, UV-B radiation, Abiotic stress, Phytohormone signaling pathway

## Abstract

**Background:**

B-box (BBX) proteins play important roles in regulating plant growth, development, and abiotic stress responses. BBX family genes have been identified and functionally characterized in many plant species, but little is known about the BBX family in blueberry (*Vaccinium corymbosum*).

**Result:**

In this study, we identified 23 *VcBBX* genes from the Genome Database for Vaccinium (GDV). These *VcBBX*s can be divided into five clades based on gene structures and conserved domains in their encoded proteins. The prediction of *cis*-acting elements in the upstream sequences of *VcBBX* genes and protein–protein interactions indicated that VcBBX proteins are likely involved in phytohormone signaling pathways and abiotic stress responses. Analysis of transcriptome deep sequencing (RNA-seq) data showed that *VcBBX* genes exhibited organ-specific expression pattern and 11 *VcBBX* genes respond to ultraviolet B (UV-B) radiation. The co-expression analysis revealed that the encoded 11 VcBBX proteins act as bridges integrating UV-B and phytohormone signaling pathways in blueberry under UV-B radiation. Reverse-transcription quantitative PCR (RT-qPCR) analysis showed that most *VcBBX* genes respond to drought, salt, and cold stress. Among VcBBX proteins, VcBBX24 is highly expressed in all the organs, not only responds to abiotic stress, but it also interacts with proteins in UV-B and phytohormone signaling pathways, as revealed by computational analysis and co-expression analysis, and might be an important regulator integrating abiotic stress and phytohormone signaling networks.

**Conclusions:**

Twenty-three *VcBBX* genes were identified in blueberry, in which, 11 *VcBBX* genes respond to UV-B radiation, and act as bridges integrating UV-B and phytohormone signaling pathways according to RNA-seq data. The expression patterns under abiotic stress suggested that the functional roles of most *VcBBX* genes respose to drought, salt, and cold stress. Our study provides a useful reference for functional analysis of *VcBBX* genes and for improving abiotic stress tolerance in blueberry.

**Supplementary Information:**

The online version contains supplementary material available at 10.1186/s12864-023-09704-8.

## Introduction

Zinc-finger transcription factors are important regulatory factors that play essential roles in plant growth, development, and responses to environmental changes [1, 2]. Zinc-finger protein family members are divided into multiple subfamilies based on their structures and functions [3]. B-box (BBX) proteins, comprising a subfamily of zinc-finger transcription factors, contain one or two conserved B-box domains at their N termini. The B-box domain is involved in protein–protein interactions and transcriptional regulation during plant signaling [4]. Some BBX proteins contain a CCT (CONSTANS, CO-like and TOC1) domain at their C termini and are referred to as CO-like (COL); the CCT domain functions in transcriptional regulation and protein targeting to the nucleus [4, 5].

*BBX* genes have been identified in various plant species, and their functions have been demonstrated at the molecular level [6, 7]. BBX proteins are mainly involved in photomorphogenesis, plant hormone signaling networks, and abiotic stress responses [6]. In Arabidopsis, BBX4, BBX11, BBX20, BBX21, BBX22, and BBX23 promoter photomorphogenesis, while BBX18, BBX19, BBX24, BBX25, BBX28, BBX29, BBX30, BBX31 and BBX32 repress photomorphogenesis in response to a wide range of light signals [8–10]. BBX4 and BBX11 promote hypocotyl elongation in red light [9, 11]. BBX11, BBX20, BBX21, BBX22 and BBX23 interact with LONG HYPOCOTYL 5 (HY5) to promote its transcriptional activation activity and positively regulate light-mediated seedling development, whereas BBX24, BBX25, BBX28, BBX29, and BBX32 interact with HY5 to form heterodimers and repress photomorphogenesis in response to light signals [10, 12–18]. For *BBX30* and *BBX31*, HY5 directly binds to their promoter to inhibit plant photomorphogenesis [17]. some studies have shown that BBX proteins also respond to UV-B radiation. For example, BBX24 from Arabidopsis represses the transcriptional activity of HY5 to negatively regulate UV-B-mediated photomorphogenic response [19]. In apple, MdCOL4/MdBBX54 (MDP0000232445) suppresses anthocyanin accumulation under UV-B and high temperature by interacting with MdHY5 to synergistically inhibit the expression of MdMYB1 [20, 21]. However, MdBBX22 (MDP0000298804) and MdBBX20 (MDP0000800387) promotes UV-B-induced anthocyanin biosynthesis through interacting with MdHY5 [22, 23]. At the same time, MdCOL11/MdBBX33 (MDP0000697407), a target of MdHY5, enhances UV-B- and temperature-induced anthocyanin biosynthesis in apple peel [24]. MdBBX37 (MDP0000157816), a negative regulator of light signaling, interacts with MdMYB1 and MdMYB9 to negatively regulate anthocyanin biosynthesis and directly binds to the promoter of *MdHY5* to relieve MdHY5-mediated hypocotyl inhibition [25]. Thus, BBX proteins regulate photomorphogenesis by interacting with MdHY5.

Several studies have indicated that BBX proteins play integrated role in phytohormones signaling pathway-mediated cellular and developmental process. For instance, in high light conditions, BBX16 integrates light and auxin to regulate plant shoot branching by the activating expression of SUPERROOT 2 (SUR2), which is a suppressor of auxin biosynthesis [26]. In gibberellin (GA) signaling pathway, BBX18 promotes hypocotyl elongation under blue light condition by regulating the expression of GA biosynthesis or metabolic genes [27]. BBX24 is a positive regulator of GA signaling by interacting and inhibiting DELLA activity [28]. In brassinosteroid (BR) signaling pathway, BBX20/BZS1, BBX28 and BBX29 negatively regulate BR signaling in *Arabidopsis* seedlings, in which BBX20 interacts with COP1 in vitro to positively regulate light signaling, and BBX28 and BBX29 physically interact with BR-ENHANCED EXPRESSION1 (BEE1), BEE2, and BEE3 to orchestrate light and BR signaling. The BBX32 interacts with PIF3 and BZR1 to negatively regulate light signaling, promote BR signaling and inhibit cotyledon opening in *Arabidopsis* [29–31]. Therefore, BBX20, BBX28, BBX29 and BBX32 mediate the crosstalk between BR and light pathways in the different ways. In abscisic acid (ABA) signaling pathway, BBX19 directly binds to promoter of ABA INSENSITIVE5 (ABI5) to negatively regulate seed germination by promoting ABA signaling [32]. In addition, OsCOL9 interacts with OsRACK1 to enhance the rice blast resistance through SA and ET signaling pathways and BBX21 downregulates the gene expression of auxin, BR and ethylene signaling pathway components under shade [7, 33]. In conclusion, BBX proteins integrate phytohormones and light signals to affect light-mediated plant growth and development [8].

In addition to photomorphogenesis and phytohormone signaling pathways, most BBX proteins also participate in plant responses to abiotic stress (cold, salt, and drought). For example, CmBBX19 from chrysanthemum (*Chrysanthemum morifolium*) interacts with CmABF3 to suppress drought tolerance, while CmBBX22 enhances drought tolerance possibly via regulating the ABA signaling pathway [34–36]. CmBBX24 improves tolerance to freezing and drought stress through influencing bioactive GA biosynthesis [37]. In Arabidopsis, BBX7 and BBX8 act downstream of CRYPTOCHROME2 (CRY2)-CONSTITUTIVELY PHOTOMORPHOGENIC 1 (COP1)-HY5 module to positively regulate blue light-dependent cold acclimation [38]. BBX24/STO binds to a Myb transcription factor homologue and enhances salt tolerance [39]. MdBBX10 from apple enhances tolerance to salt and drought stresses in Arabidopsis and involves in ABA-mediated response [40]. OsBBX11 from rice (*Oryza sativa*) regulates salt tolerance [41]. Thus, BBX proteins regulate abiotic stress response by interaction with transcript factors or phytohormone signaling pathway.

Blueberry (*Vaccinium corymbosum*) is an economically important small fruit crop that is often referred to as a “superfood” due to its high nutrient content and health benefits [42, 43]. Although *BBX* genes have been identified in various plant species via genome-wide studies based on complete plant genome sequences, a comprehensive study of *BBX* genes in blueberry has not yet been reported [21, 44–47]. A recent study showed that *VcBBX21*, *VcBBX30*, and *VcBBX32* respond to UV-B radiation [48]. However, the roles of BBX family members in abiotic stress responses in blueberry remain unknown. The release of the *Vaccinium* genus genome database offers the possibility to systematically identify and investigate the putative functions of *BBX* genes in blueberry. For *V. corymbosum*, the W8520 draft genome sequence was originally performed on March 2014, and then genome sequence was annotated and made available [49, 50]. The sequencing and assembly of the *V. corymbosum* cv. Draper genome was completed and the function was annotated on March 2019, this genome database was widely used because of high quality [51, 52].

In this study, we identified 23 blueberry *BBX* genes from the Genome Database for Vaccinium (GDV) and predicted their gene structures, as well as the physicochemical properties, evolutionary relationships, and domains of the encoded proteins. We then analyzed the expression patterns of *VcBBX* genes in different organs and the responses of the *VcBBX* genes to UV-B radiation based on transcriptome deep sequencing (RNA-seq) data. At the same time we examined the relationships between *VcBBX*s and phytohormone pathway genes under UV-B radiation based on co-expression analysis. Finally, we analyzed the expression levels of *VcBBX*s under cold, salt, and drought stress. Our results provide a foundation for further functional characterization of *VcBBX* genes in blueberry.

## Results

### Identification and physicochemical properties of *VcBBX* family members in blueberry

To identify *BBX* genes in the blueberry genome, we used Arabidopsis and apple *BBX* genes as a query to search against the GDV. We also conducted a Hidden Markov Model (HMM) search using the B-box domain (pfam00643) against the GDV. After removing short and redundant sequences, we identified 23 putative VcBBX members; detailed information is provided in Table 1 and Table S1. The VcBBX proteins ranged from 125 (VcBBX31) to 480 (VcBBX12) amino acids in length, with molecular weights ranging from 14.2 kDa (VcBBX31) to 53.0 kDa (VcBBX12). The theoretical isoelectric point (pI) was highest for VcBBX31 (8.82) and lowest for VcBBX28 (4.38). The instability index (Ii) was the lowest for VcBBX12 (43.76) and the highest for VcBBX32 (59.72) in all the VcBBX proteins, indicating that these proteins are unstable. The aliphatic index (AI) ranged from 47.70 (VcBBX29) to 71.39 (VcBBX24). The GRAVY (grand average of hydropathicity) values were negative except for VcBBX23, indicating that most VcBBXs are hydrophilic proteins. Subcellular localization of VcBBXs (predicted using the WoLF PSORT program) indicated that most VcBBX proteins are located in the nucleus. However, VcBBX6, VcBBX18, and VcBBX19 were predicted to be in the cytosol, while VcBBX23 and VcBBX29 were predicted to localize to chloroplasts, suggesting that VcBBX proteins have diverse functions.

**Table 1:** Detailed information about VcBBX proteins in blueberry

Gene ID	Gene name	Protein length (aa)	MW (kDa)	pI	Ii	AI	GRAVY	Loc
VaccDscaff13-augustus-gene-93.32	VcBBX1	350	38.8	5.00	44.87	61	–0.657	Nucleus
VaccDscaff39-augustus-gene-4.25	VcBBX6	366	39.6	6.02	46.77	63.22	–0.409	Cytosol
VaccDscaff10-augustus-gene-39.36	VcBBX7	420	45.8	5.28	58.73	61.05	–0.484	Nucleus
VaccDscaff38-processed-gene-283.17	VcBBX8	411	45.0	7.41	48.81	60.27	–0.555	Nucleus
VaccDscaff1-augustus-gene-360.27	VcBBX9	411	45.2	6.95	50.43	58.88	–0.577	Nucleus
VaccDscaff5-augustus-gene-339.39	VcBBX10	411	45.2	6.95	51.07	58.42	–0.577	Nucleus
VaccDscaff51-augustus-gene-16.34	VcBBX11	393	43.9	5.53	47.85	62.01	–0.754	Nucleus
VaccDscaff9-augustus-gene-217.24	VcBBX12	480	53.0	5.34	43.76	63.04	–0.688	Nucleus
VaccDscaff32-augustus-gene-315.40	VcBBX13	441	49.8	5.14	48.26	53.95	–0.825	Nucleus
VaccDscaff25-snap-gene-297.37	VcBBX15	386	43.6	5.70	54.22	58.65	–0.867	Nucleus
VaccDscaff32-augustus-gene-98.31	VcBBX18	210	23.2	5.91	47.27	67.81	–0.484	Cytosol
VaccDscaff30-snap-gene-112.44	VcBBX19	253	28.2	6.04	43.98	71.30	–0.337	Cytosol
VaccDscaff32-augustus-gene-95.27	VcBBX20	207	23.0	6.23	57.87	61.30	–0.575	Nucleus
VaccDscaff23-augustus-gene-352.26	VcBBX21	311	34.1	6.55	59.03	69	–0.413	Nucleus
VaccDscaff553-augustus-gene-0.13	VcBBX22	296	31.8	5.47	55.02	69.83	–0.333	Nucleus
VaccDscaff23-snap-gene-288.31	VcBBX23	200	22.3	5.33	48.73	95.5	0.009	Chloroplast
VaccDscaff3-augustus-gene-277.27	VcBBX24	245	26.9	5.15	46.32	71.39	–0.386	Nucleus
VaccDscaff9-augustus-gene-96.36	VcBBX25	240	26.5	5.28	54.82	68.33	–0.454	Nucleus
VaccDscaff6-augustus-gene-114.13	VcBBX28	242	25.9	4.38	58.13	55.58	–0.631	Nucleus
VaccDscaff15-processed-gene-164.6	VcBBX29	139	15.3	4.80	71.74	47.70	–0.736	Chloroplast
VaccDscaff6-augustus-gene-60.42	VcBBX30	227	24.0	5.00	58.39	48.55	–0.853	Nucleus
VaccDscaff38-processed-gene-234.1	VcBBX31	125	14.2	8.82	55.51	64.80	–0.271	Nucleus
VaccDscaff1-processed-gene-355.6	VcBBX32	204	22.3	5.35	59.72	53.09	–0.803	Nucleus

*MW* molecular weight; *pI* theoretical isoelectric point; *Ii* instability index; *AI* aliphatic index; *GRAVY* grand average of hydropathicity; *Loc* Predicted subcellular localization

### Phylogenetic analysis of VcBBX proteins

**Fig. 1:**
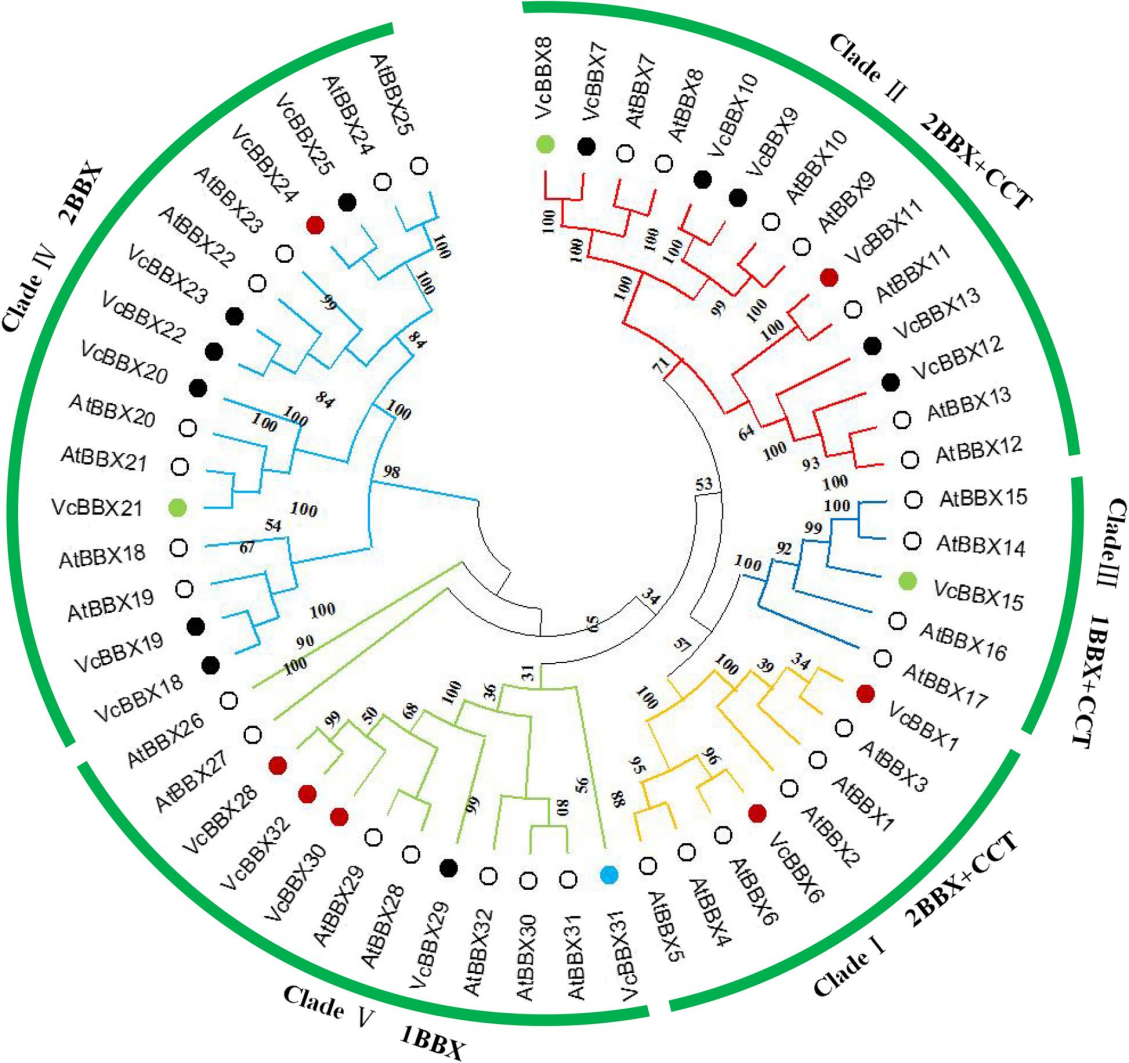
Phylogenetic analysis of BBX proteins in blueberry (*Vaccinium corymbosum*) and Arabidopsis (*Arabidopsis thaliana*). White circles represent BBX proteins from Arabidopsis. Red, green, blue, and black dots represent blueberry *BBX* genes that are upregulated, downregulated, up- or downregulated, and not regulated by UV-B radiation, respectively

**Fig. 2:**
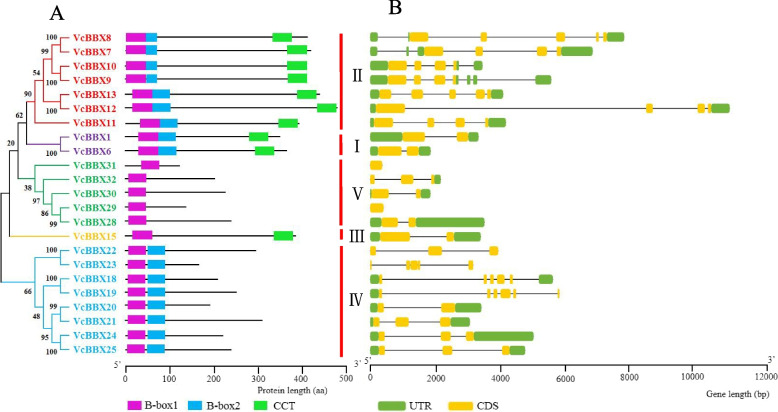
Phylogenetic relationships and structural analysis of blueberry BBXs. **A** Phylogenetic analysis of VcBBX proteins: different colors represent different groups and structures of VcBBX proteins. The pink, blue, and green boxes represent B-box1, B-box2, and CCT domains, respectively. **B** Structures of *VcBBX* genes. Dark green boxes, orange boxes, and black lines represent UTRs, exons, and introns, respectively

To investigate the evolutionary relationships of VcBBX family members, we reconstructed a phylogenetic tree based on the amino acid sequences of the 23 blueberry VcBBXs and 32 Arabidopsis AtBBXs (Fig. 1; Table S2). The VcBBXs were divided into five clades and named based on phylogenetic analysis and previous studies in Arabidopsis [5]. VcBBX1 and VcBBX6 were grouped into clade I with AtBBX1–6, and VcBBX7–13 were grouped into clade II with AtBBX7–13. Proteins in these two clades contain B-box1 and B-box2 domains, as well as a CCT domain. VcBBX18–25 were grouped into clade IV with AtBBX18–25; proteins in this clade contained B-box1 and B-box2 domains. Clade III proteins, comprising VcBBX15 and AtBBX15–17, contain B-box1 and CCT domains. Clade V consists of VcBBX28–32 and AtBBX26–32, which only contain a B-box1 domain (Figs. 1 and 2A).

To explore the structures of the 23 *VcBBX* genes in blueberry, we obtained exon/intron information about them based on their coding and genomic sequences from the GDV. The number of exons ranged from one to five. *VcBBX29* and *VcBBX31* contain no introns or untranslated regions (UTRs), and *VcBBX23* also lacks a UTR. Similar results were found in BBX families of tomatoes and peaches [45, 53]. Similarly, *VcBBX* genes with similar gene structures clustered into the same clade. For example, members of clade II contain four or five exons, and members of clade I harbor two exons (Fig. 2B).

### Analysis of phytohormone- and abiotic stress–related *cis*-acting elements

**Fig. 3:**
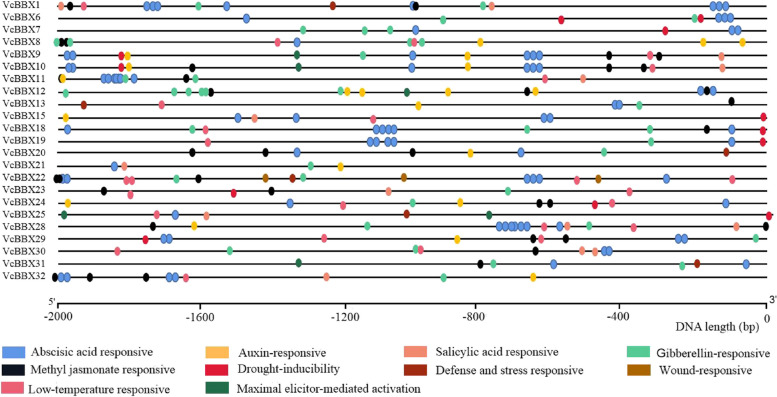
Diagram of the predicted regulatory *cis*-elements involved in phytohormone and abiotic stress responses in the *VcBBX* promoters. The black line below the diagram indicates the lengths of the *VcBBX* promoters. The different colored symbols represent *cis*-acting elements with different functions, as described below the diagram

To better understand the functions of *VcBBX* genes, we collected their promoter sequences 2000 bp upstream from the ATG start codon and predicted the *cis*-acting elements involved in phytohormone and abiotic stress responses using the PlantCARE online tool (Fig. 3). The *VcBBX* promoters contained various *cis*-acting elements related to phytohormones, such as ABA-, auxin-, SA-, GA-, and methyl jasmonate (MeJA)-responsive elements. These promoters also contained many abiotic stress-related elements, especially drought-inducible and low-temperature-responsive elements.

### VcBBX protein interactions

**Fig. 4:**
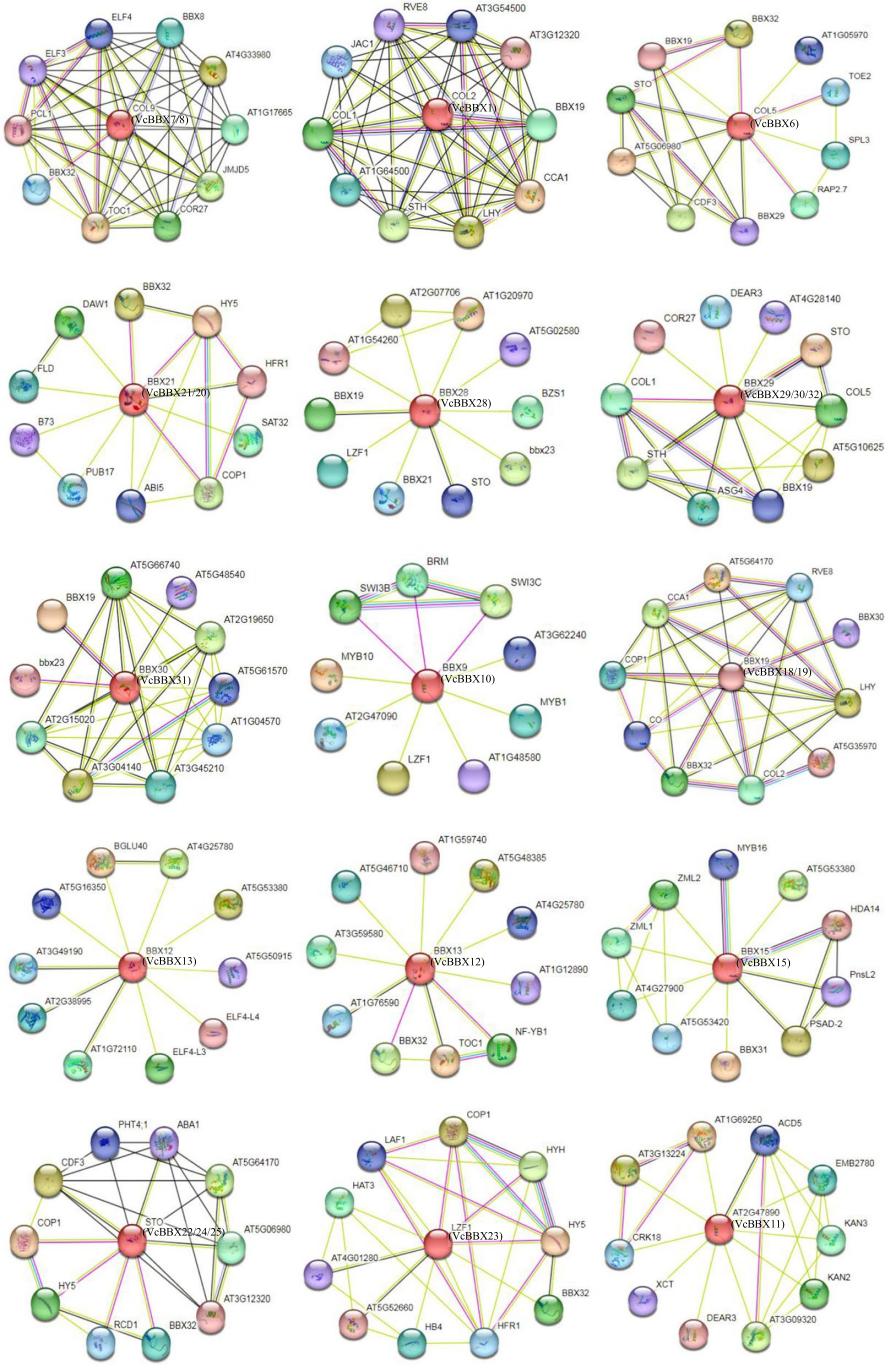
Protein interaction networks predicted for 23 VcBBX proteins based on their orthologs in Arabidopsis using the online tool STRING. The red balls indicate VcBBX proteins; the names of the orthologs in blueberry are shown in brackets. The other colored balls represent individual VcBBX interactors. The blue and purple lines represent known interactions from curated databases and those that were experimentally determined, respectively. The green, red, and blue lines represent predicted interactions from gene neighborhood, gene fusions, and gene co-occurrence, respectively. The yellow-green, black, and blue lines represent text mining, co-expression, and protein homology, respectively. The acronyms define proteins that have been reported in Arabidopsis

To predict the functions of the VcBBXs, we assembled a protein interaction network for each member using their orthologs from Arabidopsis (Fig. 4). The 23 VcBBX proteins in blueberry correspond to 15 BBX/COL proteins in Arabidopsis. The acronyms and full names of the interactors are provided in Table S3. These proteins interacted not only with BBX proteins (STH, BZS1, BBX32, BBX8, CO, BBX9, BBX31, and COL1), but also with other proteins such as transcription factors (MYB: MYB1, MYB10, MYB16; basic helix-loop-helix [bHLH]: HFR1; bZIP: HY5, HYH; zinc-finger: CDF3), an E3 ubiquitin-protein ligase (COP1), and proteins involved in the circadian clock (XCT, PCL1, LHY, ELF3, ELF4, ELF4-LIKE3, and ELF4-LIKE4). VcBBX29/30/32 (BBX29 ortholog), VcBBX11 (At2g47890 ortholog), VcBBX6 (COL5 ortholog), and VcBBX22/24/25 (COL2 ortholog) were predicted to interact with proteins in the ethylene signaling pathway (DEAR3, RAP2.7, TOE2, and RCD1), while VcBBX20/21 (BBX21 ortholog) and VcBBX22/24/25 (STO/BBX24 ortholog) likely interact with proteins in the ABA signaling pathway (ABI5 and ABA1). We also observed that VcBBX29/30/32 (BBX29 ortholog) and VcBBX7/8 (COL9 ortholog) are predicted to interact with COR27, which is involved in cold stress responses. These results indicate that VcBBX proteins may be participated in plant growth and development, as well as plant responses to abiotic stress and phytohormone signaling pathways.

### The organ-specific expression pattern analysis of VcBBX genes in blueberry

**Fig. 5:**
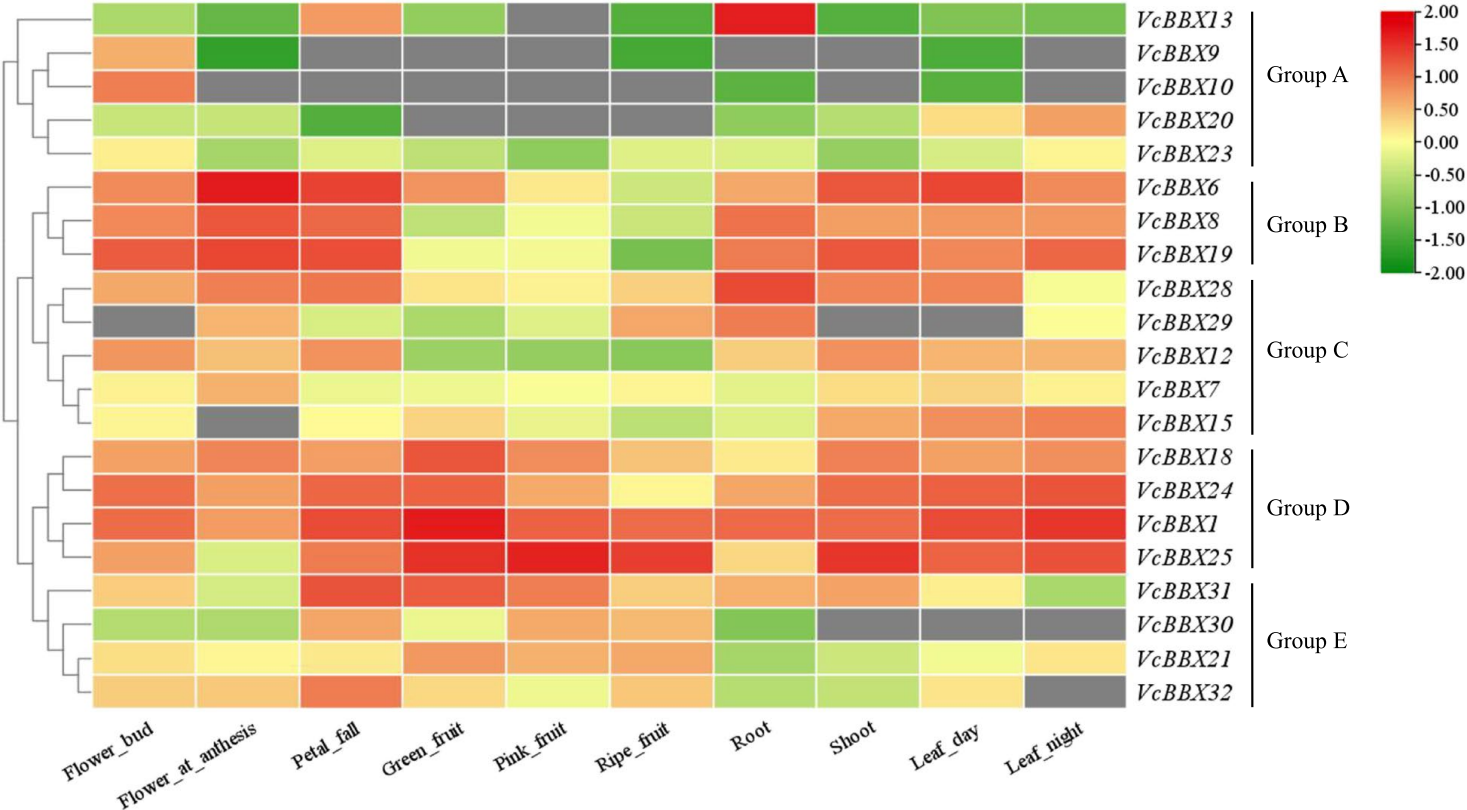
Transcript profiling of *VcBBX* genes in various organs based on log_10_ (FPKM) values from RNA-seq data. The color scale is shown on the right, block with green indicated low expression levels, while red indicated high expression levels. The gray block indicated that the gene was not expressed

To provide clues to the putative roles of the *VcBBX* genes in blueberry development, the expressions of VcBBX family genes were analyzed in flower bud, flower at anthesis, petal fall, green fruit, pink fruit, ripe fruit root, shoot, leaf at day, and leaf at night from transcriptome sequencing results and the heatmap of organic-specific expressions was drawn based on log_10_ (FPKM) values (Fig. 5). The VcBBXs were divided into five groups and same groups shared similar expression patterns. The *VcBBX13*, *VcBBX9*, *VcBBX10*, *VcBBX20* and *VcBBX23* were clustered in group A because they did not expressed or showed low expression in some organs. The members of group B (*VcBBX6*, *VcBBX8* and *VcBBX9*) and group C (*VcBBX28*, *VcBBX29*, *VcBBX12* and *VcBBX7*) showed low expression during fruit development, however, the expression levels of *VcBBX* members from group B are higher than that from group C in root, shoot, leaf, and flower. The *VcBBX18*, *VcBBX24*, *VcBBX1*, and *VcBBX25* were clustered in the group D and showed high expression in all the organs and the expression of *VcBBX31*, *VcBBX30*, *VcBBX21*, and *VcBBX32* from group E were higher in the flowers and fruits than in the root, shoot and leaf. These results indicated that most blueberry *VcBBX* genes showed organ-specific expression pattern, potentially suggesting the functional divergence of *VcBBX* genes. The *VcBBX18*, *VcBBX24*, *VcBBX1*, *VcBBX25*, *VcBBX31*, *VcBBX30*, *VcBBX21*, and *VcBBX32* may play important role in regulation of flower and fruit development.

### *VcBBX* gene expression in response to UV-B radiation

**Fig. 6:**
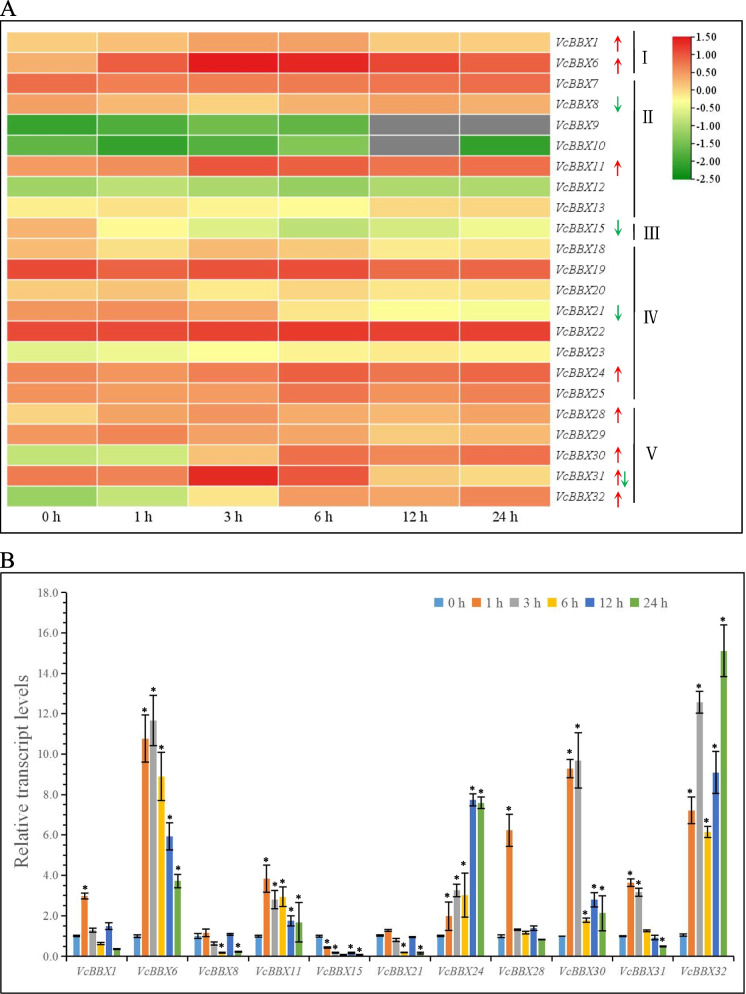
Expression analysis of *VcBBX* genes under UV-B radiation. **A** Transcript profiling of *VcBBX* genes under UV-B radiation based on log_10_ (FPKM) values from RNA-seq data. The color scale is shown on the right, block with green indicated low expression levels, while red indicated high expression levels. The gray block indicated that the gene was not expressed. **B** Gene expression patterns of *VcBBX* genes under UV-B radiation using qRT-PCR analysis. Values are means ± SD from three independent biological replicates, each with three technical replicates; Statistically significant differences were assessed using Student’s t-test (* *p* < 0.05)

To reveal the roles of *VcBBX*s in plant responses to abiotic stress, we downloaded RNA-seq data from the BioProject database, which were obtained from blueberry calli treated for 0, 1, 3, 6, 12, and 24 h with UV-B radiation [52]. Eleven *VcBBX*s were responsive to UV-B radiation, including *VcBBX8*, *VcBBX15*, and *VcBBX21* (from cladesII, III, and IV, respectively), which were downregulated; and *VcBBX1*, *VcBBX6*, *VcBBX11*, *VcBBX24*, *VcBBX28*, *VcBBX30*, and *VcBBX32* (from various clades), which were upregulated. *VcBBX31* was upregulated at 3 and 6 h and downregulated at 12 and 24 h of UV-B treatment (Fig. 6A and Table S4). These results suggest that VcBBXs from different clades might participate in the same regulatory pathways. In all the differentially expressed *VcBBX* genes, *VcBBX6* expression was upregulated during UV-B treatment and the value of log_10_ (FC) reached 2.09 (1 h), 4.01 (3 h), 3.61 (6 h), 2.84 (12 h), and 2.16 (24 h) under UV-B treatment. The expression of *VcBBX30* also was upregulated from 3 h onwards, reaching the highest level at 24 h (5.16 for log_10_ (FC) value). However, *VcBBX15* was downregulated from 1 h onwards, reaching the lowest level (-3.22 for log_10_ (FC) value) at 6 h in expression levels.

To validate the accuracy and reliability of the RNA-seq data under UV-B radiation, expression levels of eleven differentially expressed *VcBBX* genes under UV-B radiation for 0, 1, 3, 6, 12, and 24 h were analyzed by the RT-qPCR (Fig. 6B). Consistent with RNA-Seq data, the expression of *VcBBX1*, *VcBBX6*, *VcBBX11*, *VcBBX24*, *VcBBX28*, *VcBBX30*, and *VcBBX32* significantly upregulated and that of *VcBBX8*, *VcBBX15*, and *VcBBX21* significantly downregulated under UV-B treatment compared to the 0-h control. Furthermore, the relative expression levels of VcBBX6 significantly increased 10.76-, 11.66-, 8.89-, 5.9–3, and 3.71-fold at 1, 3, 6, 12, and 24 h of UV-B treatment relative to the 0 h treatment, respectively. The expression of *VcBBX30* also was significantly upregulated during UV-B treatment and reached the highest level at 3 h (9.69-fold compared to the 0-h control). We also found that *VcBBX24* and *VcBBX32* also was significantly upregulated and increased more than seven-fold during UV-B treatment compared to the 0-h control. RNA-seq data and RT-qPCR analysis showed that *VcBBX6*, *VcBBX24*, *VcBBX30* and *VcBBX32* maybe play important roles under UV-B radiation.

### Correlations between *VcBBX* genes and phytohormone pathway genes under UV-B radiation

**Fig. 7:**
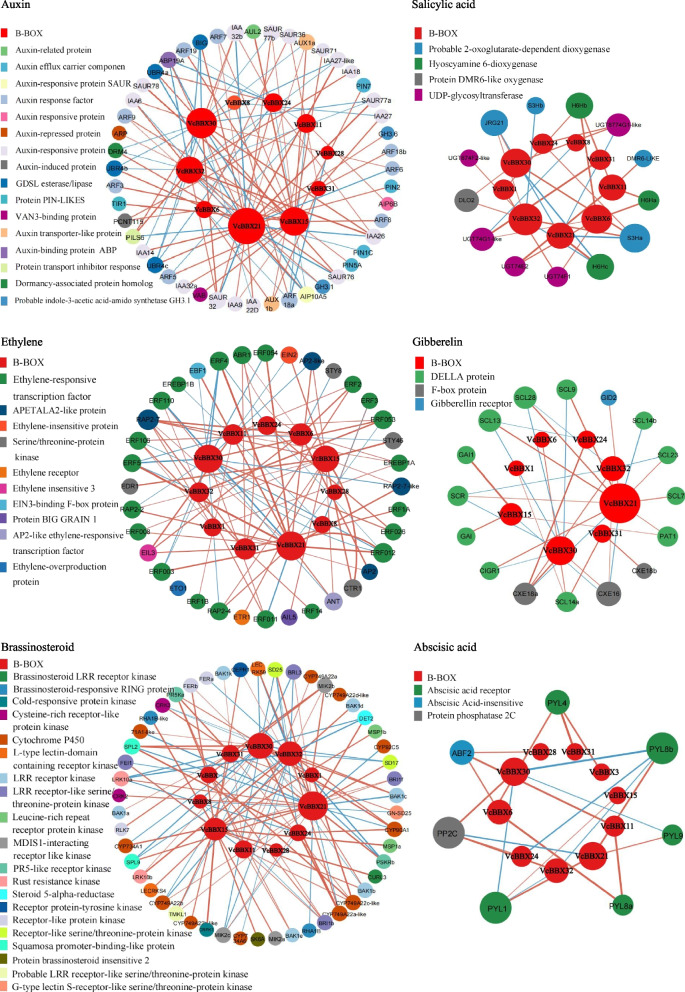
Co-expression network analysis of DEGs between *VcBBX*s and plant hormone signaling–related genes under UV-B radiation. Red lines indicate positive correlations; blue lines indicate negative correlations. The thickness of the line represents the degree of correlation. The size of the circle represents the number of related genes

The *VcBBX* promoter sequences contain various *cis*-acting elements related to phytohormones. Thus, we searched for differentially expressed genes (DEGs) from phytohormone pathways in RNA-seq data set of UV-B treatment and performed Pearson’s correlation coefficient (*r*) analysis between the *VcBBX*s and DEGs in various phytohormone pathways according to FPKM values (Table S5). Forty-eight genes from the auxin pathway showed a significant correlation in their expression levels with various *VcBBX* genes. Specifically, 27 auxin pathway genes showed a significant correlated in their expression with that of *VcBBX21*, 20 with *VcBBX30*, and 18 with *VcBBX15*; however, *VcBBX28* expression levels were only significantly correlated with those of *AUXIN RESPONSE FACTOR 18a* (*ARF18a*). Forty-one genes in the ethylene pathway exhibited a significant correlation with *VcBBX* gene expression. Of these, *VcBBX21* expression was significantly correlated with the largest number of genes (17) from the ethylene pathway, followed by *VcBBX30* (16) and *VcBBX15* (14), while *VcBBX28* expression was only significantly correlated with that of *ETHYLENE-RESPONSE FACTOR 3* (*ERF3*). The expression levels of 50 BR pathway genes were significantly correlated with those of *VcBBX*s. *VcBBX21* was correlated with the most genes (25), followed by *VcBBX30* (20), *VcBBX32* (18), and *VcBBX15* (15). Finally, the expression of 13, 16, and 7 genes was significantly correlated with that of *VcBBX* genes in the SA, GA, and ABA pathways, respectively, with *VcBBX21*, *VcBBX30*, and *VcBBX32* expression being significantly correlated with the most number of genes from these three phytohormone pathways. At the same time, *VcBBX24* also was significantly correlated with genes from auxin (7), GA (2), ABA (2), Ethylene (5), BR (6) and SA (2) under UV-B radiation (Fig. 7; Table S5).

### Expression analysis of *VcBBX* genes under abiotic stress

**Fig. 8:**
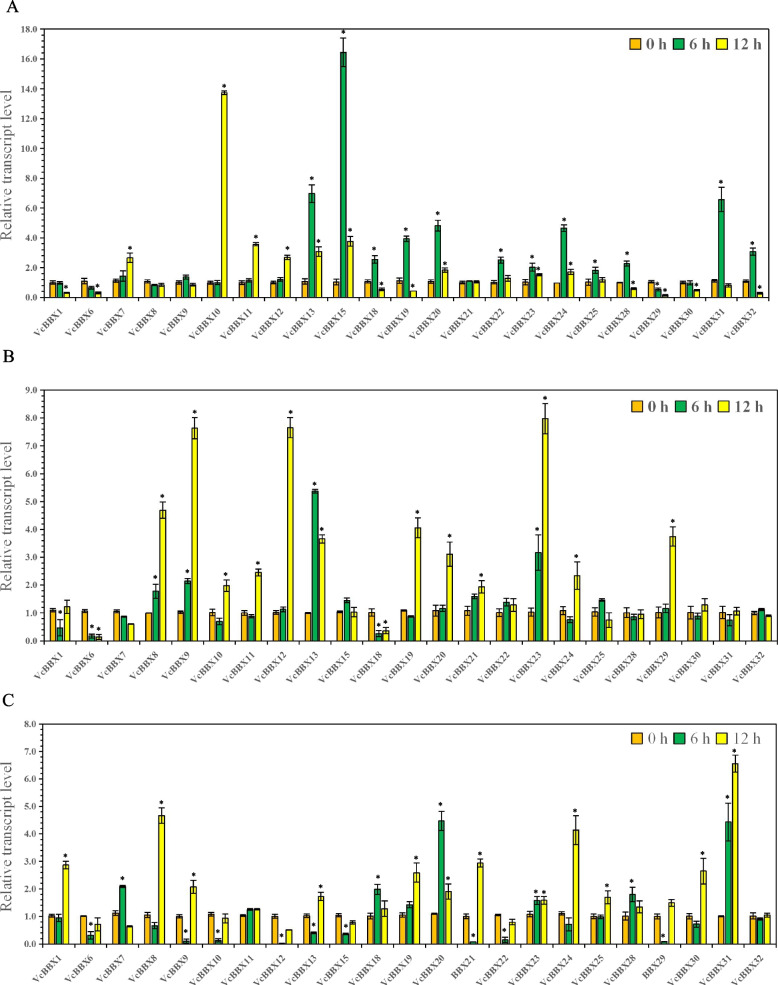
Expression patterns of the 23 *VcBBX* genes in response to three abiotic stresses. **A** *VcBBX* expression under drought stress (20% PEG-6000). **B** *VcBBX* expression under salt stress (200 mM NaCl). **C** *VcBBX* expression under cold stress (5℃). All treatments were performed for 6 or12 h. Values are means ± SD from three independent biological replicates, each with three technical replicates; Statistically significant differences were assessed using Student’s t-test (* *p* < 0.05)

To further explore the roles of VcBBXs in plant responses to abiotic stress, we evaluated the expression patterns of the 23 *VcBBX* genes in the leaves of plants subjected to drought, salt, or cold stress by RT-qPCR (Fig. 8). The *VcBBX* genes exhibited different expression patterns in response to drought. The expression of most *VcBBX* genes (16/23) was significantly upregulated in response to drought treatment, especially *VcBBX10*, *VcBBX13*, *VcBBX15*, *VcBBX19*, *VcBBX20*, *VcBBX24*, and *VcBBX31*. However, the expression of *VcBBX1*, *VcBBX6*, *VcBBX29*, and *VcBBX30* was significantly downregulated in response to this stress, and *VcBBX8*, *VcBBX9*, and *VcBBX21* were not responsive to 6 or 12 h of drought stress (Fig. 8A). Fifteen *VcBBX* genes were responsive to salt stress, with twelve *VcBBX* genes being upregulated and three being downregulated. The relative expression levels of *VcBBX8*, *VcBBX9*, *VcBBX11–13*, *VcBBX19*, *VcBBX20*, *VcBBX23*, *VcBBX24*, and *VcBBX29* increased more than two-fold following 6 or 12 h of salt stress treatment compared to the 0-h control (Fig. 8B).

The expression of 21 *VcBBX* genes responded to cold treatment. The twelve *VcBBX* genes were upregulated and six were downregulated at 6 or 12 h of cold treatment. In which, the transcript levels of *VcBBX9*, *VcBBX13*, and *VcBBX21* rapidly decreased at 6 h of cold treatment and increased at 12 h of treatment. The transcript levels of *VcBBX1*, *VcBBX7–9*, *VcBBX19–21*, *VcBBX24*, *VcBBX30*, and *VcBBX31* increased more than two-fold at 6 or 12 h of treatment compared to the control (Fig. 8C). Many *VcBBX* genes also showed different responses to different stresses; for example, *VcBBX22* was induced by drought, inhibited by cold, and did not respond to salt stress. Importantly, most *VcBBX* genes were induced by drought, cold, and salt stress, indicating that the roles of *VcBBX* genes in abiotic stress are conserved in plants. In summary, *VcBBX19*, *VcBBX20*, and *VcBBX24* were signifcantly upregulated by drought, salt, and cold stress and increased more than two-fold at 6 or 12 h of treatment relative to the 0 h treatment, indicating that *VcBBX19*, *VcBBX20*, and *VcBBX24* may be involved in a variety of abiotic stresses.

## Discussion

### Structure of VcBBX genes and evolutionary analyses

The *BBX* gene family has been identified in many plants, including fruit crops. However, the number of BBX family members differs among plant species. To date, 32 *BBX* genes have been identified in Arabidopsis, 64 in apple, 21 in strawberry (*Fragaria vesca*), 25 in pear (*Pyrus communis*), 24 in grapevine (*Vitis vinifera*) and peanut (*Arachis duranensis*), 22 in peach (*Prunus persica*) and 15 in sweet cherry (*Prunus avium*) [5, 21, 33, 46, 54–57]. In this study, after deleting short and redundant sequences, we identified 23 full length *BBXs* in blueberry. Thus, blueberry contains fewer *BBX* genes than most other plant species. These differences may be due to differences in genome size as well as tandem and segmental duplication events among plant species. However, the composition of conserved domains in the encoded BBX proteins of blueberry is similar to that of other species. We identified four types of blueberry BBX proteins: those with two tandem B-boxes and one CCT domain, those with one B-box domain and one CCT domain, those with two tandem B-boxes, and those with one B-box domain [5, 46, 53].

Most studies have divided BBX family members into five clades, and BBX proteins with the same types of conserved domains clustered together. For example, BBX proteins from pear, Arabidopsis, purple false brome (*Brachypodium distachyon*), rice, and black cottonwood (*Populus trichocarpa*) were divided into five groups: group I or II proteins contain B-box1, B-box2, and CCT domains; group III proteins contain B-box1 and CCT domains; group IV proteins contain B-box1 and B-box2 domains; and group V proteins contain B-box1 domains [55]. We obtained the same results for blueberry BBX family members. At the same time, the number of exons in *BBX* genes belonging to the same clade were similar in blueberry and other species [45, 55]. These results suggest that *BBX* families in different species might share a common ancestor and similar evolutionary patterns to adapt to the environment.

### The function predication of VcBBX genes based on organ-specific expression analysis

Gene expression analysis in essential for providing clues for functional prediction [44, 45]. In this study, VcBBX genes showed distinct expression patterns among organs. In which, VcBBX1, 6, and 8 showed a high expression in flower bud, flower at anthesis, and petal fall and they also have one CCT domain, Some study showed that BBX proteins with a CCT domain play critical role in flowering, suggesting that VcBBX1, 6, and 8 play important roles in flowering [5, 58]. Most *VcBBX* genes come from groups IV and V, including *VcBBX18*, *VcBBX21*, *VcBBX24*, *VcBBX25*, and *VcBBX30-32*, have higher expression in petal fall, green fruit, pink fruit and ripe fruit than other *VcBBX* genes. The groups IV and V contain one or two B-box domains without CCT domain. At same time, *VcBBX21*, *VcBBX24*, and *VcBBX30-32* response to UV-B radiation, and UV-B radiation involve in color of flower and fruit [48]. In apple, MdBBX20, MdBBX22, MdBBX33 and MdBBX54 of group IV with two B-box domains without CCT domain regulate UV-B-induced anthocyanin biosynthesis and MdBBX37 of group V with one B-box domain without CCT domain also regulates anthocyanin biosynthesis [20, 22–24]. In pear, PpBBX16, PpBBX18 and PpBBX21, with two B-box domains without CCT domain, regulate anthocyanin accumulation [59, 60]. The previous study showed that *VcBBX30* and *VcBBX32* co-expressed with the possible anthocyanin biosynthesis gene *VcMYBA2* and *VcMYB114* under UV-B radiation [48]. Thus, *VcBBX21*, *VcBBX24*, and *VcBBX30-32*, especially *VcBBX30* and *VcBBX32*, may regulate UV-B-induced anthocyanin biosynthesis. Above all we speculated that B-box domains might play critical role in anthocyanin biosynthesis. In this study, we also found that *VcBBX18*, *VcBBX24*, *VcBBX1*, and *VcBBX25* were high expression in all the organs indicating that they have various roles in development of blueberry different organs.

### The function predication of VcBBX genes under UV-B radiation

Light is a major environmental factor that regulates physiology and development in plants, such as photomorphogenesis, flowering, and anthocyanin accumulation. As a component of natural light, UV-B also regulates photomorphogenic responses. Inhibition of hypocotyl growth and promotion of anthocyanin accumulation are the most obvious effects of excess UV-B exposure [61–65]. BBX proteins play essential roles in these UV-B-mediated responses, and most *BBX* genes are induced by UV-B light in Arabidopsis [66]. AtBBX24 interacts with COP1 and HY5 in UV-B-induced inhibition of hypocotyl elongation, and AtBBX31 regulates UV-B-mediated photomorphogenesis in a HY5-dependent manner [19, 67, 68]. In apple, MdBBX22 promotes UV-B-induced anthocyanin biosynthesis by interacting with MdHY5, and MdCOL4 interacts with MdHY5 to synergistically inhibit anthocyanin accumulation under UV-B radiation [22, 23].

In the current study, 11 of the 23 *VcBBX* genes from five different clades responded to UV-B radiation, consisting of seven *VcBBX* genes, three downregulated genes, and one that was upregulated and then downregulated during UV-B treatment. Thus, most *VcBBX* genes are likely involved in UV-B-induced photomorphogenic responses; however, their specific functions require further study. The predicted protein interaction networks based on orthologous BBXs of Arabidopsis showed that VcBBX18–25 interact with HY5, HY5 HOMOLOG (HYH), and COP1. The previous study showed that VcBBX30 and VcBBX32 co-expressed with the VcHY5, and VcHY5 co-expressed with COP1 under UV-B radiation [48]. Moreover, *VcBBX21*, *VcBBX24*, *VcBBX30*, and *VcBBX32* were regulated by UV-B radiation. Thus, *VcBBX21*, *VcBBX24*, *VcBBX30*, and *VcBBX32* might interact with HY5, HYH, or COP1 to regulate UV-B-induced photomorphogenesis or anthocyanin accumulation.

### The VcBBX genes may involved in phytohormone signaling pathways under UV-B radiation

BBX proteins play important roles in phytohormone signaling pathways [33, 34]. We determined that all sequences upstream of *VcBBX* genes contain ABA-, auxin-, SA-, GA-, and MeJA-responsive elements. The expression levels of 11 *VcBBX*s were significantly correlated with those of genes from the auxin, ethylene, BR, SA, GA, and ABA signaling pathways during UV-B radiation, as revealed by co-expression analysis based on RNA-seq data (Figs. 3 and 4). Thus, most blueberry VcBBX proteins might function in phytohormone signaling pathways. Several studies have shown that BBX proteins are involved in the crosstalk between light and phytohormone signaling during plant development [27, 37, 69]. However, little is known about how *BBX* genes coordinate with genes from phytohormone signaling pathways to regulate UV-B-induced physiology and development in plants. AtBBX24 interacts with DELLA proteins in the GA pathway to regulate UV-B-induced photomorphogenesis in Arabidopsis [28, 70]. We determined that 11 *VcBBX*s respond to UV-B radiation, and their expression was significantly correlated with that of many genes involved in phytohormone signaling pathways, especially the auxin, ethylene, and BR pathways. The expression of *VcBBX21*, *VcBBX30*, *VcBBX32*, and *VcBBX15* was significantly correlated with that of most genes involved in phytohormone signaling pathways under UV-B radiation. In addition, VcBBX6, VcBBX11, VcBBX20, VcBBX21, VcBBX22, VcBBX24, VcBBX25, VcBBX29, VcBBX30, and VcBBX32 maybe interact with proteins in the ethylene or ABA signaling pathway, however protein interaction tests are needed for further confirmation. Thus, we propose that VcBBXs may serve as bridges integrating UV-B signaling and phytohormone signaling pathways in blueberry.

### The expression pattern of VcBBX genes under abiotic stresses

*BBX* genes are involved in various stress responses in many plant species [21, 34]. For example, overexpressing *AtBBX24* (also named *SALT TOLERANCE* [*AtSTO*]) improved salt tolerance in Arabidopsis, and heterologous expression of *CmBBX22* from chrysanthemum (*Chrysanthemum morifolium*) improved drought tolerance in this plant [35, 39]. Most *BBX* genes also respond to salt, drought, and cold stress [35, 39, 47, 71]. Our study showed that 20 *VcBBX* genes respond to drought stress, 15 to salt stress, and 21 to cold stress, demonstrating that most *VcBBX* genes generally respond to abiotic stress. *VcBBX13*, *VcBBX19, VcBBX20*, *VcBBX23*, and *VcBBX24* were upregulated and *VcBBX6* was downregulated in response to all three stresses, suggesting that these genes play major roles in abiotic stress responses (Fig. 8).

*VcBBX10*, *VcBBX13*, *VcBBX15*, *VcBBX19*, *VcBBX20*, *VcBBX24*, and *VcBBX31* were most strongly upregulated by drought stress among all *VcBBX* genes, suggesting that they might play major roles in plant responses to drought stress. *VcBBX1*, *VcBBX7–9*, *VcBBX19–21*, *VcBBX24*, *VcBBX30*, and *VcBBX31* were most strongly upregulated by cold treatment. Among their encoded proteins, VcBBX7, VcBBX8, and VcBBX30 were predicted to interact with COR27, suggesting that these proteins play major roles in regulating the cold stress response. Moreover, *VcBBX8*, *VcBBX9*, *VcBBX11–13*, *VcBBX19*, *VcBBX20*, *VcBBX23*, *VcBBX24*, and *VcBBX29* were mainly regulated by salt stress. In conclusion, VcBBXs play important roles in plant responses to abiotic stress. *VcBBX24* responds to UV-B radiation, drought stress, salt stress, and cold stress, and VcBBX24 interacts with UV-B signaling pathway proteins (COP1 and HY5), an ABA signaling pathway protein (ABA-INSENSITIVE 1 [ABA1]), and an ethylene signaling pathway protein (RADICAL-INDUCED CELL DEATH1 [RCD1]). Similarly, AtBBX24 (VcBBX24 ortholog) is involved in plant responses to salt stress and UV-B radiation in Arabidopsis [39]. Thus, we propose that VcBBX24 is an important component of the abiotic stress regulatory network in blueberry.

## Conclusions

In this study, we identified 23 putative *VcBBX* genes in the blueberry genome and classified them into five clades based on phylogenetic analysis and the conserved motifs of their encoded proteins. Analysis of *cis*-acting elements and prediction of protein interactions suggested that *VcBBX* genes participate in diverse phytohormone pathways and abiotic stress responses. The organ-specific expression analysis showed that *VcBBXs* played various roles in blueberry development. Analysis of RNA-seq data and co-expression analysis indicated that *VcBBX* genes function in plant responses to UV-B radiation and act as bridges between UV-B signaling and phytohormone signaling pathways. Furthermore, most *VcBBX* genes respond to drought, salt, and cold stress. These results provide valuable information for further analysis of the functions of *VcBBX* genes to guide breeding efforts for improved abiotic stress resistance in blueberry.

## Materials and methods

### Identification of *BBX* genes in the blueberry genome

To identify *BBX* genes in blueberry, two different procedures were used. First, the sequences of *BBX* genes from Arabidopsis (*Arabidopsis thaliana*) and apple (*Malus domestica*) were downloaded from The Arabidopsis Information Resource database (TAIR, https://www.arabidopsis.org/) and the Genome Database for Rosaceae (GDR, https://www.rosaceae.org/), respectively, and used as a query against the Genome Database for *Vaccinium corymbosum* cv. Draper V1.0 genome sequence (GDV, https://www.vaccinium.org/). Second, Hidden Markov Model (HMM) searches were performed in the GDV using the B-box domain (pfam00643) from the Pfam database (http://pfam.xfam.org/). The potential *VcBBX* genes in blueberry were investigated using the online programs UniProt (https://ww.uniprot.org/), SMART (http://smart.embl-heidelberg.de/), and CDD (https://www.ncbi.nlm.nih.gov/Structure/cdd/wrpsb.cgi), and genes without BBX domains in their encoded proteins were removed. Finally, a list of VcBBX genes encoding complete BBX domains was obtained by deleting shorter and redundant sequences.

The VcBBX members were named based on clustering with Arabidopsis proteins and functional annotations from a manually annotated and reviewed protein sequence databaset (Swiss-Prot). The molecular weight (MW), theoretical isoelectric point (pI), instability index (Ii), aliphatic index (AI), and grand average of hydropathicity (GRAVY) of the VcBBX proteins were calculated using the online program ExPASy (https://web.expasy.org/protparam/), and the subcellular localization of each VcBBX protein was predicted using the online program WoLF PSORT (https://www.genscript.com/wolf-psort.html?src=leftbar).

### Phylogenetic analysis

The phylogenetic trees were reconstructed in MEGA X using the neighbor-joining method. Bootstrap analysis was carried out with 1000 replicates [72]. Arabidopsis sequences were downloaded from NCBI (https://www.ncbi.nlm.nih.gov/). The phylogenetic trees were divided into different clades refered studies on Arabidopsis [5].

### Analysis of gene structures and conserved domains

The cDNA sequences and the corresponding untranslated regions (UTRs), exons, and introns of *VvBBX* family members were downloaded from the GDV and gene structure was drawn with TBtools software (v1.120) [73]. Conserved domains including B-box and CCT domains were identified using the online programs UniProt, SMART, and CDD.

### Analysis of *cis*-acting regulatory elements in the promoters of *VvBBX* genes

The 2000-bp upstream sequences of *VcBBX* genes were downloaded from the GDV and submitted to the online program PlantCARE (https://bioinformatics.psb.ugent.be/webtools/plantcare/html/) to predict *cis*-acting elements. The *cis*-acting elements related to phytohormone and abiotic stress responses were retained and mapped along the presumptive promoters.

### Construction of a protein interaction network

The VcBBX protein sequences were submitted to the online program STRING (https://string-db.org/). The orthologous proteins from Arabidopsis with the highest bit scores were chosen to predict the interacting proteins and to construct the protein–protein interaction networks; *Arabidopsis thaliana* was used as the reference organism.

### Analysis organ-specific expression pattern of VcBBX genes

The expression profiles of *VcBBX* gene from blueberry cultivar ‘Draper’ in different organs including flower bud, flower at anthesis, petal fall, green fruit, pink fruit, ripe fruit, root, shoot, leaf at day and leaf at night, were downloaded from the BioProject database in the NCBI repository (https://www.ncbi.nlm.nih.gov/bioproject/PRJNA494180).

### The expression pattern of *VcBBX* genes under UV-B radiation and co-expression network construction

The expression profiles of *VcBBX* genes and phytohormone signaling-related pathway genes under UV-B radiation from blueberry calli were downloaded from the BioProject database in the NCBI repository (https://www.ncbi.nlm.nih.gov/bioproject/PRJNA831018). Pearson’s correlation coefficient (*r*) analysis was performed between differentially expressed *VcBBX*s and genes in phytohormone signaling-related pathways under UV-B radiation (for 0, 1, 3, 6, 12, or 24 h) according to fragments per kilobase of transcript per million fragments mapped (FPKM) values using SPSS 19.0 software. Genes with a *p*-value ≤ 0.05 were considered to be significantly correlated. The co-expression networks were visualized based on *r* value of significant correlation using Cytoscape v3.9.1 software. The heatmaps of blueberry *VcBBX* genes based on log_10_ (FPKM) values in different organs and under UV-B radiation were drawn with TBtools software (v1.120) [73].

### Stress treatments and reverse-transcription quantitative PCR

Six-month-old blueberry cultivar ‘Northland’ plants from tissue culture were grown in a growth chamber at 25 °C with 70% relative humidity under a 16-h-light/8-h-dark photoperiod. The first to third fully expanded leaves were collected from plants following treatment with salt (200 mM), cold (5 °C), or drought (20% [w/v] polyethylene-glycol) for 6 or 12 h, using samples collected at time 0 (0 h) as control. Total RNA was extracted from the leaves using a Plant RNA Extraction Kit (Sangon Biotech, Shanghai, China). The cDNAs were synthesized using PrimeScript™ RT Master Mix (TaKaRa, Japan). Reverse-transcription quantitative (RT-qPCR) analysis was performed using an ABI 7900HT Real-time PCR system. The relative expression levels of the *VcBBX*s were calculated using the 2^–∆∆Ct^ method and RT-qPCR results were normalised to 0 h. The glyceraldehyde-3-phosphate dehydrogenase housekeeping gene (*GAPDH*; GenBank accession no. AY123769) was used as the reference gene. Primer sequences for qPCR are shown in Table S6. All experiments were carried out with three independent biological replicates, and three technical replicates were performed for each biological replicate. One-way ANOVA was used to assess the differences in expression levels of *VcBBX* genes in plants subjected to the same stress for different periods, and Tukey’s test was used to identify significant differences using SPSS 19.0 software.

### Supplementary Information

**Additional file 1: Table S1.** Homologous Arabidopsis and blueberry genes of the BBX family. **Table S2.** Gene ID of VcBBXs in blueberry and AtBBXs in Arobidopsis. **Table S3.** The acronyms and corresponding full name for predicted VcBBX interactors. **Table S4.**  Information of BBX genes under UV-B radiation. **Table S5.** Pearson's correlation coefficients (r) of expression levels between B-Box and plant hormone signlaling genes under UV-B radiation. **Table S6.** Primers used in this study.

## Data Availability

All data generated or analysed during this study are included in this article [and its supplementary information files]. The raw sequencing data from this study has been deposited in in the NCBI repository https://www.ncbi. nlm.nih.gov/bioproject/PRJNA892908 for UV-B treatment and https://www.ncbi.nlm.nih.gov/bioproject/PRJNA494180 for organ-specific expression. BioSample: SAMN31399479 (UV-B 0 h), BioSample: SAMN31399480 (UV-B 1 h), SAMN31399481 (UV-B 3 h), SAMN31399482 (UV-B 6 h), SAMN31399483 (UV-B 12 h), and SAMN31399484 (UV-B 24 h) for UV-B treatment; SAMN10438841 (flower_bud), SAMN10438842 (flower_at_anthesis), SAMN10438849 (petal_fall), SAMN10438843 (green_fruit), SAMN10438850 (pink_fruit), SAMN10438851 (ripe_fruit), SAMN10438853 (shoot), SAMN10438852 (root), SAMN10438845 (leaf_night), and SAMN10438844 (leaf_day) for organ-specific expression.
